# HGTphyloDetect: facilitating the identification and phylogenetic analysis of horizontal gene transfer

**DOI:** 10.1093/bib/bbad035

**Published:** 2023-02-08

**Authors:** Le Yuan, Hongzhong Lu, Feiran Li, Jens Nielsen, Eduard J Kerkhoven

**Affiliations:** Department of Biology and Biological Engineering, Chalmers University of Technology, Kemivägen 10, SE-412 96 Gothenburg, Sweden; Novo Nordisk Foundation Center for Biosustainability, Chalmers University of Technology, Kemivägen 10, SE-412 96 Gothenburg, Sweden; School of Life Sciences and Biotechnology, Shanghai Jiao Tong University, 200240 Shanghai, China; Department of Biology and Biological Engineering, Chalmers University of Technology, Kemivägen 10, SE-412 96 Gothenburg, Sweden; Department of Biology and Biological Engineering, Chalmers University of Technology, Kemivägen 10, SE-412 96 Gothenburg, Sweden; BioInnovation Institute, Ole Måløes Vej 3 DK-2200 Copenhagen, Denmark; Department of Biology and Biological Engineering, Chalmers University of Technology, Kemivägen 10, SE-412 96 Gothenburg, Sweden; Novo Nordisk Foundation Center for Biosustainability, Chalmers University of Technology, Kemivägen 10, SE-412 96 Gothenburg, Sweden

**Keywords:** horizontal gene transfer, phylogenetic analysis, gene transmission, evolution analysis

## Abstract

**Background:**

Horizontal gene transfer (HGT) is an important driver in genome evolution, gain-of-function, and metabolic adaptation to environmental niches. Genome-wide identification of putative HGT events has become increasingly practical, given the rapid growth of genomic data. However, existing HGT analysis toolboxes are not widely used, limited by their inability to perform phylogenetic reconstruction to explore potential donors, and the detection of HGT from both evolutionarily distant and closely related species.

**Results:**

In this study, we have developed HGTphyloDetect, which is a versatile computational toolbox that combines high-throughput analysis with phylogenetic inference, to facilitate comprehensive investigation of HGT events. Two case studies with *Saccharomyces cerevisiae* and *Candida versatilis* demonstrate the ability of HGTphyloDetect to identify horizontally acquired genes with high accuracy. In addition, HGTphyloDetect enables phylogenetic analysis to illustrate a likely path of gene transmission among the evolutionarily distant or closely related species.

**Conclusions:**

The HGTphyloDetect computational toolbox is designed for ease of use and can accurately find HGT events with a very low false discovery rate in a high-throughput manner. The HGTphyloDetect toolbox and its related user tutorial are freely available at https://github.com/SysBioChalmers/HGTphyloDetect.

## Background

Horizontal gene transfer (HGT), also known as lateral gene transfer, refers to the exchange of genetic material between disparate groups of organisms other than from parent to offspring [1]. This has been recognized as significantly contributing to adaptive evolution, disease emergence and metabolic shifts that can act across various species [2–4]. An important mechanism of HGT occurrence is transformation, which is the active import and inheritable integration of naked DNA from the extracellular environment [5]. The probability of transformation depends on various physiological determinants, of which the efficiency of the DNA uptake machinery is a major factor in the rate of transformation [3]. HGT events have a particularly high frequency of occurrence in prokaryotes, and it is one of the main mechanisms contributing to genetic variation and thus evolution [6]. Although HGT occurs relatively less frequent in microbial eukaryotes compared to prokaryotes, it remains an important contributor to the evolution of eukaryotic genomes, especially in facilitating the gain of adaptive functions [7].

Although the mechanism of HGT is very complex and often occurs at different rates across prokaryotes and eukaryotes, there are still a few computational approaches available to predict HGT events. For example, HGT-Finder is a phyletic distribution-based tool that calculates a horizontal transfer index and probability value for each query gene, but, unfortunately, this software is no longer available for download [8]. HGTector is a customized pipeline for genome-wide detection of HGT events based on sequence homology search hit distribution statistics, but lacks in systematic phylogeny analysis to explore the underlying mechanism of horizontally acquired gene transmission [9]. Another method called AvP can automate the robust identification of potential HGT events within a phylogenetic framework [10]; however, the phylogenetic trees produced by this approach are not of high quality and it is uncertain whether HGT events from evolutionarily closely related species could be detected or not. Consequently, although a few efforts have been made for the identification of HGT events, current HGT detection approaches do have various limitations and are not widely suitable to the entire HGT community. High-performance computational tools that are able to robustly identify HGT events in a high-throughput and user-friendly manner are lacking and urgently needed.

To this end, we therefore developed HGTphyloDetect, an open-source computational toolbox to investigate HGT events by combining with phylogenetic inference. In this work, we adopted high-throughput algorithms for the identification of HGT events no matter whether the target horizontally acquired genes are from evolutionarily distant species or from closely related species, showcasing its versatility. Furthermore, we applied this new bioinformatics software to the whole genomes of two species (*Saccharomyces cerevisiae* and *Candida versatilis*). It can be found that HGTphyloDetect presents its great performance by comparing the predicted horizontally acquired genes with those published in previous studies. More importantly, the HGTphyloDetect toolbox enables the generation of high-quality phylogenetic trees to further facilitate the navigation of potential donors and detailed elucidation of a feasible path of gene transmission.

## Implementation

### Detection of HGT from evolutionarily distant organisms

**Figure 1:**
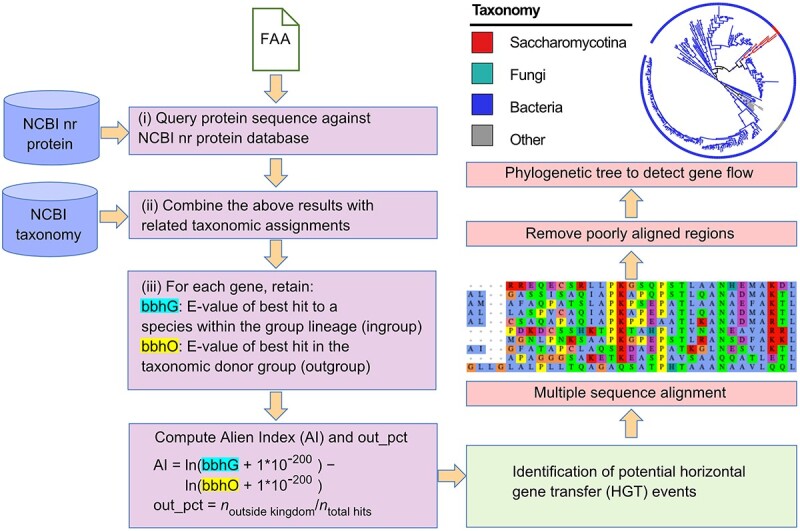
Overview of the HGTphyloDetect pipeline for automated identification of HGT events from evolutionarily distant organisms (e.g. prokaryote to eukaryote).

To identify potential genes that have been horizontally acquired from evolutionarily distant organisms (e.g. prokaryote to eukaryote), we defined a robust and phylogeny-based approach as shown in Figure 1. First, the National Center for Biotechnology Information (NCBI) non-redundant (nr) protein database is queried for a specific gene or several genes of interest by BLASTP. These BLASTP hits are then parsed to retrieve associated taxonomic information from the NCBI taxonomy database based on the toolkit ETE v3 [11]. With this information, Alien Index (AI) scores are calculated as follows:

(1) \begin{equation*} \mathrm{AI}=\mathit{\ln}\left(\mathrm{bbhG}+1\ast{10}^{-200}\right)-\mathit{\ln}\left(\mathrm{bbhO}+1\ast{10}^{-200}\right) \end{equation*}

Here, bbhG and bbhO represent the E-values of the best BLAST hit in ingroup and outgroup lineages, respectively. The ingroup lineage is defined as the species inside of the kingdom, but outside of the subphylum. The outgroup lineage is defined as all species outside of the kingdom. The Alien Index mathematical formula used here has previously been defined in an impactful study by Gladyshev et al., who found that AI ≥ 45 is a good indicator of foreign origin [12]. In addition, to remove inaccurate results of HGT identification, for each gene, the percentage of hits from the outgroup that have different taxonomic species names are calculated as follows:

(2) \begin{equation*} \mathrm{out}\_\mathrm{pct}={n}_{\mathrm{outside}\ \mathrm{kingdom}}/{n}_{\mathrm{total}\ \mathrm{hits}} \end{equation*}

Finally, those genes with AI ≥ 45 and out_pct ≥ 90% are assumed as likely HGT candidates from evolutionarily distant species [4, 12, 13]. The threshold value for out_pct is adopted based on an influential work by Shen et al., who evaluated the parameter and its great power in removing inaccurate HGT events [4]. While providing default AI and out_pct parameters, users can also easily define different values in HGTphyloDetect to tune the accuracy of their predictions.

### Detection of HGT from closely related organisms

Although the above workflow is powerful to detect HGT events from evolutionarily distant organisms, we have also constructed a complementing workflow for automated detection of HGT events from more closely related organisms (e.g. eukaryote to eukaryote; see Supplementary Figure S1 for details), thereby greatly expanding the versatility of this computational toolbox.

For this workflow (Supplementary Figure S1), several steps are carried out to obtain potential horizontally acquired genes as follows: (i) BLASTP process is performed against the NCBI nr protein database by taking a collection of genes as input and related taxonomic information for each gene hit is retrieved; (ii) at the first round of preliminary screening, genes with a best hit in the kingdom lineage (excluding the recipient subphylum lineage) and a bitscore ≥100 are screened; (iii) HGT index (or comparative similarity index) is calculated as the bitscore of the best hit in a potential donor (the species inside of the kingdom, but outside of the subphylum) divided by the bitscore of the best hit in the recipient (the species inside of the subphylum), where all genes with HGT index ≥50% are retained, as this indicates that these genes match well to other genes in potential donors; (iv) for each gene, the percentage of species from potential donors (the species inside of the kingdom, but outside of the subphylum) that have different taxonomic species names is determined, if this is ≥80% (this threshold value is adopted considering that most gene hits should belong to the taxonomy category of potential donors if the query gene is horizontally acquired from other evolutionarily close species), then the gene is retained. These parameter threshold values listed above are mainly selected based on some previously published studies [13–15]; meanwhile, users can also easily define different threshold values to fine-tune their analysis. Finally, those remaining genes are defined as horizontally acquired genes from closely related organisms.

### Construction of the phylogenetic analysis pipeline

To corroborate the accurate identification of HGT genes by their AI values, as described above, we extended HGTphyloDetect with a phylogenetic analysis pipeline. First, the top 300 homologs with different taxonomic species names are selected from the BLASTP hits for each query sequence. HGTphyloDetect then aligns these homologs with MAFFT v7.310 [16] using default settings for multiple sequence alignment, whereas ambiguously aligned regions are removed with trimAl v1.4 using its ‘-automated1’ option [17]. To ensure robust and high-quality phylogenetic trees, phylogenetic trees are constructed from these alignments using IQ-TREE v1.6.12 [18] with 1000 ultrafast bootstrapping replicates, whereas bootstrap scores of the internal branches of trees are calculated based on IQ-TREE v1.6.12. Subsequently, each phylogenetic tree is rooted at the midpoint using ape v5.4-1 [19] and phangorn v2.5.5 [20]. Finally, the resulting phylogenies are visualized using iTol v5 (https://itol.embl.de/) [21] to assess the mode of transmission of each gene.

## Results and discussion

### Basic usage and applications of HGTphyloDetect

HGTphyloDetect (available from https://github.com/SysBioChalmers/HGTphyloDetect). is relatively easy to use, as users only need to prepare a FASTA file containing both protein identifier and protein sequence as input. The large NCBI nr protein and taxonomy databases that are used in the pipeline are accessed remotely on demand, precluding the need to download these large databases. The installation with only few dependencies and a detailed user tutorial is all well documented (available from https://github.com/SysBioChalmers/HGTphyloDetect/blob/master/User%20tutorial.pdf).

A user-friendly example for HGT detection is provided with HGTphyloDetect. This example follows a typical scenario of HGTphyloDetect application, where the aim is to identify HGT events and potential donors for either one gene or all genes in one species, or even up to all genes in hundreds of species. The scalability of HGTphyloDetect allows it to be readily included as part of a larger analysis workflow. For example, Lu and colleagues integrated HGT analysis and genome-scale metabolic models (GEMs) approaches to reveal the main driving force behind metabolic innovation for expanding substrate usage across more than 300 yeast species [13, 22]. In particular, HGTphyloDetect can be used in HGT detection not only for prokaryotes but also for eukaryotes. This means that large-scale genome-wide HGT analysis in prokaryotic genomes and eukaryotic genomes is allowed to be investigated at the same time via HGTphyloDetect.

### Testing the performance of HGTphyloDetect

**Figure 2:**
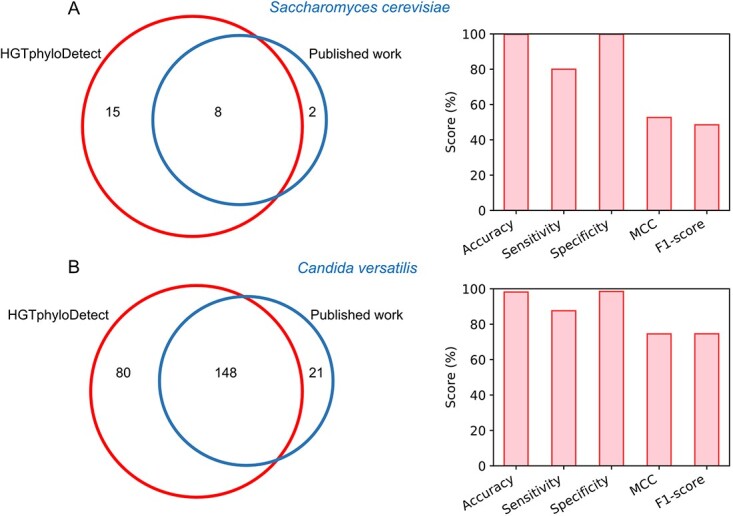
Case studies of the HGTphyloDetect computational toolbox. (**A**) Comparison of the number of horizontally acquired genes in *S. cerevisiae* identified by the HGTphyloDetect toolbox and reported by previously published work. (**B**) Performance of the HGTphyloDetect toolbox in *C. versatilis* via comparing the predicted horizontally acquired genes with those in previously published work.

To evaluate the prediction performance of HGTphyloDetect, we applied this toolbox to two species (*S. cerevisiae* and *C. versatilis*) that have manually curated HGT events described in previously published works, allowing benchmarking of our approach [4, 23]. For *S. cerevisiae*, 10 horizontally acquired genes from bacteria have been reported by previous work [23]. By running HGTphyloDetect for all (more than 6000) genes in *S. cerevisiae* with the default parameters, we were able to identify 23 HGT gene candidates from bacteria (Supplementary Table S1), of which 8 gene candidates were previously reported (Figure 2A), that is, YNR058W (BIO3), YDR540C, YJL217W, YKL216W (URA1), YFR055W, YOL164W (BDS1), YMR090W and YNR057C (BIO4), respectively. The remaining 15 genes that HGTphyloDetect identified were not previously reported to be linked to HGT, although the Alien Index, out_pct and E-values reported here (Supplementary Table S1) provide strong evidence for their bacterial origin. That the bacterial origin of these genes in the model organism *S. cerevisiae* had not previously been suggested can testify for the previous lack of sufficient data and suitable computational approaches. As a result, HGTphyloDetect showed a very low false positive rate and high accuracy (Figure 2A).

In addition, we were able to predict that 27 *S. cerevisiae* genes have been obtained from horizontal transfer from more evolutionarily closely related fungal species (Supplementary Table S2). Of those 27 genes, only 5 were predicted to have been donated from the *Taphrinomycotina*, *Ustilaginomycotina* and *Agaricomycotina* lineages, whereas the remaining 22 genes were potentially horizontally acquired from the widely distributed *Pezizomycotina* subphylum (Supplementary Table S2), which is composed of a vast number of filamentous species. As existing computational tools had not been able to identify HGT events between eukaryotes, this is the first study to systematically predict genes that have potentially been obtained in the well-known *S. cerevisiae* through horizontal transfer from closely related species.

For *C. versatilis*, more horizontally acquired genes (169 in total) have been reported, rendering it suitable to further benchmark HGTphyloDetect. Here, by applying the high-throughput pipeline into the whole (more than 5000) genes of *C. versatilis* with the default parameters, we were able to identify 148 out of 169 genes as horizontally acquired genes (Figure 2B). Subsequently, several standard evaluation metrics comprising sensitivity, specificity and accuracy were adopted to assess the prediction performance of HGTphyloDetect based on true positive, true negative, false positive and false negative, in which true positive indicates that the manually curated horizontally acquired gene in peer-reviewed literature was predicted as a horizontally acquired gene by HGTphyloDetect. From the calculation, the accuracy, sensitivity and specificity values were found to be 98.16%, 87.57% and 98.49%, respectively (Figure 2B). Therefore, HGTphyloDetect again showed its high-quality performance when comparing the predicted HGT gene candidates with those previously reported in literature [4].

### Comparison with other existing approaches

**Figure 3:**
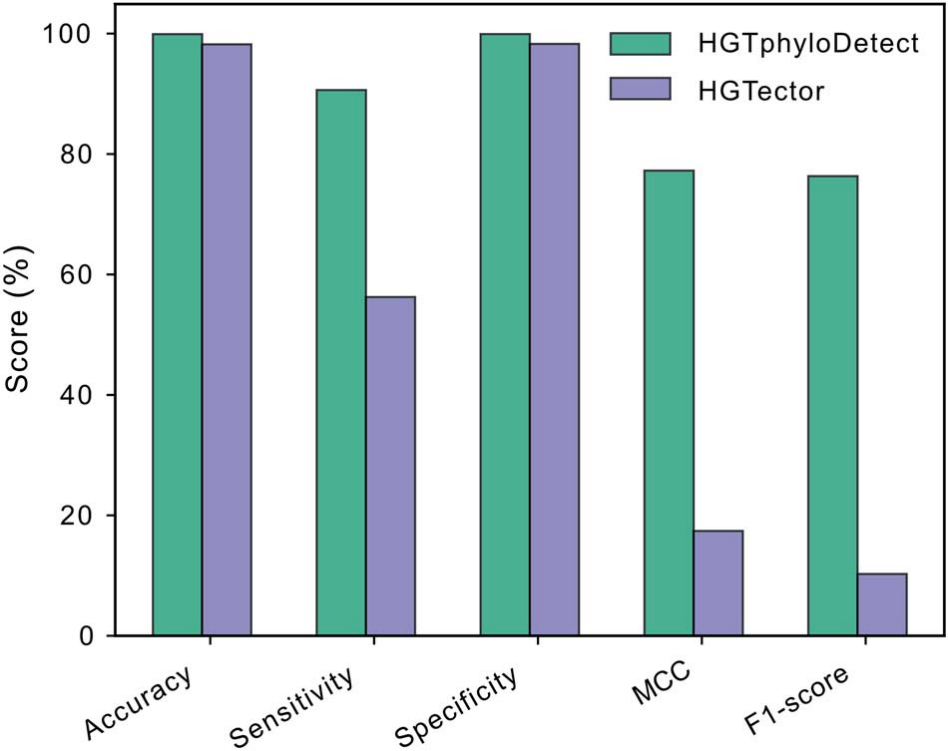
Comparison of the HGT detection performance between HGTphyloDetect and other existing computational tools, i.e. HGTector.

We further evaluated HGT detection performance by comparing HGTphyloDetect with other existing computational tools, e.g. the HGTector toolbox that can also be used to detect HGT events in a high-throughput manner [9]. For this, we adopted the benchmark dataset published by the Rokas group [4], in which they systematically analyzed and manually inspected the identification of HGT events across over 300 yeast species. Due to the large computations required for HGT identification, we randomly selected three yeast species for which HGT events were identified in that study (*Lipomyces kononenkoae*, *Kluyveromyces lactis*, *Lachancea fermentati*), including more than 15 000 unique genes in total. HGT detection workflows were run for all those genes with HGTphyloDetect and HGTector, and various evaluation metrics were used for comparison, e.g. accuracy, sensitivity, specificity, etc. HGTphyloDetect had somewhat higher accuracy and specificity than HGTector (Figure 3), but more significantly, the sensitivity, Matthews correlation coefficient (MCC) and F1-score from HGTphyloDetect was much higher compared with HGTector (Figure 3).

### Phylogenetic analysis example via HGTphyloDetect

**Figure 4:**
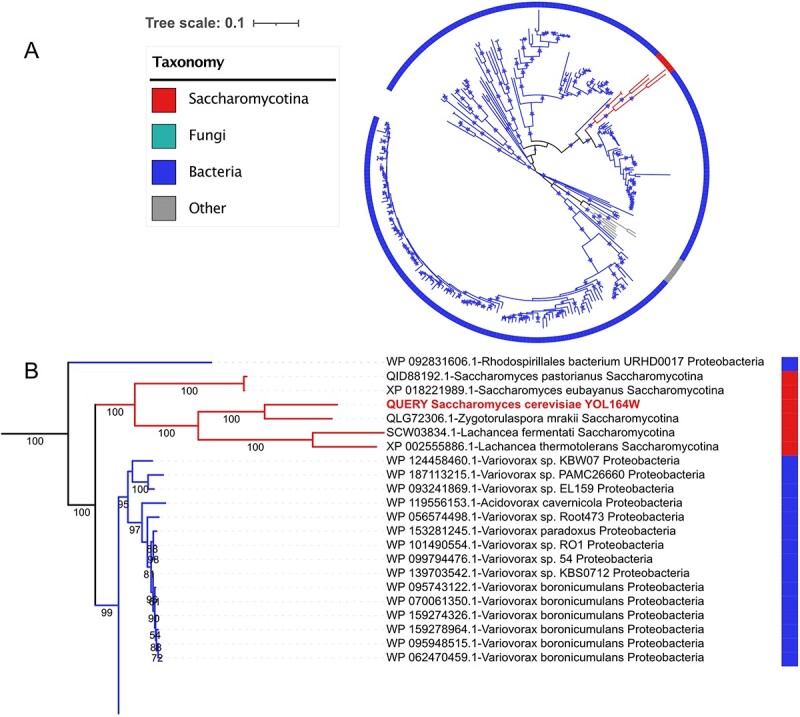
An example of an HGT event from prokaryote to eukaryote shown by the phylogenetic tree. (**A**) ML phylogeny of a protein YOL164W in *S. cerevisiae*. Branches with bootstrap support higher than 80% are shown by a star. (**B**) The detailed phylogenetic tree represents pruned ML phylogeny depicting the phylogenetic relationship between this protein from the *Saccharomycotina* subphylum and its close relatives from other bacteria.

The most accurate and golden-standard approach for the identification of horizontally acquired genes is gene-by-gene phylogenetic analysis [24], which compares the target gene phylogeny with similar genes from other species. As a case study for the phylogenetic analysis with HGTphyloDetect, we navigated the maximum likelihood (ML) phylogeny of YOL164W, an important protein in *S. cerevisiae* enabling alkyl sulfatase activity and arylsulfatase activity that is assumed to be acquired through HGT [23]. To probe the evolutionary trajectory of this protein and its potential origin, a detailed phylogenetic tree was constructed based on the wrapped pipeline in HGTphyloDetect, for which the ML phylogeny was reconstructed by utilizing the top homolog hits obtained using the protein sequence of YOL164W as a query. With HGTphyloDetect, we noted that the high-quality phylogenetic tree for the YOL164W protein could be clearly generated (Figure 4A). From the phylogenetic tree, it becomes obvious that YOL164W has highly likely been horizontally acquired from a bacterial species. Although inspection of the pruned phylogenetic tree can aid to identify the potential bacterial donor and allows to explore the phylogenetic relationship between this protein and its close relatives from proteobacteria, we found that all the internal branches close to the query protein have bootstrap scores of more than 95%, indicating the significance of HGT events detected by HGTphyloDetect (Figure 4B). With this showcase, phylogenetic analysis with HGTphyloDetect enables the investigation of gene transferring mechanism for potential HGT events.

### Challenges and future perspectives

Given the rapid increase in newly sequenced genome data, we observe a great demand for a software solution capable of identifying HGT events for the further investigation of gene variation and evolution. The high-quality performance of HGTphyloDetect that we demonstrated here render it able to meet wide biological application demands from various fields, e.g. interpreting the pathogen phenotype in fungi [25], analyzing antibiotic resistance determinants in bacteria [26], new functionality exploration in the gut microbiome [27]. HGTphyloDetect can now enable the investigation of these different phenotypes on a large scale, which could help to explore which genes are likely acquired from HGT and whether these HGT genes are involved in the generation of these important phenotypes. Even though the HGTphyloDetect toolbox yields great performance for the identification of HGT events, various challenges remain. Critically, although HGTphyloDetect can provide strong computational evidence for potential horizontally transferred genes, it remains elusive how to conduct a systematic experimental test for HGT validation. To address this, it is advisable to only give credence to genes with strong AI, out_pct and E-value scores. Currently, HGTphyloDetect cannot be applied to detect mosaic genes that receive genetic material from different donors, but combining other available tools (e.g. the method developed by Vladimir Makarenkov et al. [28]) would enable the investigation of HGT events on mosaic genes. In addition, although HGTphyloDetect now enables novel analyses, it could benefit from performance improvements that would make it more suitable to perform very large scale analyses.

As an alternative approach, it is conceivable that machine learning could be utilized to predict horizontally acquired genes. Machine learning has been applied and shown its great power in solving various gene- or protein-related problems such as in prediction of gene essentiality [13], gene expression [29] and enzyme turnover numbers [30, 31]. Indeed, few efforts have already been made in this direction, as the deep learning model DeepHGT can accurately find HGT insertion sites on genomes based on the sequence pattern [32]. However, although the use of machine learning approaches in HGT research has a strong potential, it is hampered by its reliance on large high-quality datasets that are obtained from experiments. The availability of such datasets is sparse in HGT research, although, in addition, most machine learning approaches use black-box models for prediction [33] that are not suitable to detect potential donors and origin genomes in contrast to HGTphyloDetect.

HGTphyloDetect will continue to be developed and maintained on GitHub together with its users and researchers in the HGT ecosystem. We hope that HGTphyloDetect can become a standardized toolbox for identifying HGT and its underlying mechanisms in the large scientific community.

## Conclusions

In summary, we created HGTphyloDetect, a versatile toolbox to automatically identify potential HGT events via a high-throughput approach combined with phylogenetic analysis. It is applicable to detect the probable mechanisms underlying gain-of-function that are highly relevant to evolutionary biology, systems biology, synthetic biology and many other domains.

## Authors’ contributions

L.Y., H.Z.L. and E.J.K. designed the research. L.Y. performed the research. L.Y., H.Z.L., F.R.L., J.N. and E.J.K. analyzed the data. H.Z.L. and E.J.K. supervised the work. All authors interpreted the results, discussed, drafted and approved the final manuscript.

## Data Availability

HGTphyloDetect is freely available, open source and distributed on GitHub. The computational software, installation, examples and documentation file for user tutorial are available in the repository: https://github.com/SysBioChalmers/HGTphyloDetect.

**Key points**

- HGTphyloDetect is a comprehensive toolbox to facilitate the identification of horizontal gene transfer events, no matter whether the horizontally acquired genes are from evolutionarily distant species or from close species.
- Two case studies with *S. cerevisiae* and *C. versatilis* demonstrate the ability of HGTphyloDetect to identify horizontally acquired genes with high accuracy.
- In-depth phylogenetic analysis facilitates the navigation of potential donors and detailed elucidation of a feasible path of gene transmission.

## Supplementary Material

supplementaryFile_bbad035

## Author Biographies

**Le Yuan** is a PhD student at Chalmers University of Technology, Sweden. His research interests include bioinformatics, comparative genomics and machine learning.

**Hongzhong Lu** is now a tenure-track associate professor at Shanghai Jiao Tong University (SJTU), China. His research interests include bioinformatics, systems biology and synthetic biology. He has published more than 20 academic papers in top journals, including Nature Communications, Molecular System Biology, etc.

**Feiran Li** is a postdoc researcher at Chalmers University of Technology, Sweden. Her research interests include systems biology, computational biology and machine learning.

**Jens Nielsen** is a professor at Chalmers University of Technology, Sweden. Prof. Nielsen is member of several academies, including the National Academy of Engineering and National Academy of Science in the USA, Chinese Academy of Engineering, the Royal Swedish Academy of Science, the Royal Danish Academy of Science and Letters. He has published more than 850 papers that have been cited more than 100 000 times (current H-index 144).

**Eduard J Kerkhoven** is a senior researcher and group leader at Chalmers University of Technology, Sweden. His research interests include systems biology, metabolic engineering and synthetic biology. He has published more than 40 academic papers in top journals, including Nature Catalysis, Nature Energy, Nature Communications, PNAS, etc.
