# Supplementary material for: HGTphyloDetect: facilitating the identification and phylogenetic analysis of horizontal gene transfer
Source: Brief Bioinform. 2023 Feb 8;24(2):bbad035. doi: 10.1093/bib/bbad035 (PMC10025432; doi:10.1093/bib/bbad035)
Supplement: supplementaryFile_bbad035 [file supplementaryfile_bbad035.docx]

**Supplementary information for HGTphyloDetect: facilitating the identification and phylogenetic analysis of horizontal gene transfer**

Le Yuan^1,2^, Hongzhong Lu^3*^, Feiran Li^1^, Jens Nielsen^1,4^ and Eduard J Kerkhoven^1,2*^

^1^ Department of Biology and Biological Engineering, Chalmers University of Technology, Kemivägen 10, SE-412 96 Gothenburg, Sweden

^2^ Novo Nordisk Foundation Center for Biosustainability, Chalmers University of Technology, Kemivägen 10, SE-412 96, Gothenburg, Sweden

^3^ School of Life Sciences and Biotechnology, Shanghai Jiao Tong University, 200240, Shanghai, China

^4^ BioInnovation Institute, Ole Måløes Vej 3, DK2200 Copenhagen N, Denmark

* Corresponding author:

Hongzhong Lu, Tel: +86-021 3420 4126; Email: hongzhonglu@sjtu.edu.cn

Eduard J Kerkhoven, Tel: +46-031 772 1000; Email: eduardk@chalmers.se

**Supplementary figure 1**. Overview of the HGTphyloDetect workflow for automated prediction of horizontal gene transfer (HGT) from close organisms (e.g., eukaryote to eukaryote).

**Supplementary table 1**. Gene candidates for horizontal transfer from outside of fungi in *Saccharomyces cerevisiae* via high-throughput identification.

| **Gene** | **Alien index** | **Out_pct** | **E value** | **Donor taxonomy** |
| --- | --- | --- | --- | --- |
| YNR058W | 278.87 | 1.0000 | 7.7e-122 | Bacteria/Proteobacteria |
| YPL273W | 105.74 | 1.0000 | 1.2e-46 | Bacteria/Proteobacteria |
| YKL084W | 68.29 | 1.0000 | 2.2e-30 | Bacteria/Bacteroidetes |
| YLL060C | 212.86 | 1.0000 | 3.6e-93 | Bacteria/Proteobacteria |
| R0020C | 460.52 | 1.0000 | 2.1e-215 | Bacteria/Proteobacteria |
| YDR540C | 154.09 | 1.0000 | 1.2e-67 | Bacteria/Firmicutes |
| YKL070W | 46.29 | 1.0000 | 7.9e-21 | Bacteria/Proteobacteria |
| YJL217W | 171.36 | 1.0000 | 3.8e-75 | Bacteria/Proteobacteria |
| YKL152C | 228.54 | 1.0000 | 5.6e-100 | Bacteria/Actinobacteria |
| YIR042C | 112.13 | 0.9877 | 4.8e-117 | Bacteria/Proteobacteria |
| YKL216W | 307.06 | 1.0000 | 4.4e-134 | Bacteria/Firmicutes |
| YFR055W | 319.80 | 1.0000 | 1.3e-139 | Bacteria/Proteobacteria |
| YOL056W | 90.82 | 1.0000 | 3.6e-40 | Bacteria/Actinobacteria |
| YDL021W | 90.80 | 1.0000 | 3.7e-40 | Bacteria/Actinobacteria |
| YGR234W | 276.39 | 1.0000 | 9.2e-121 | Bacteria/Proteobacteria |
| YKL218C | 300.90 | 1.0000 | 2.1e-131 | Bacteria/Proteobacteria |
| YKL107W | 149.23 | 0.9423 | 5.4e-76 | Bacteria/Proteobacteria |
| YOL164W | 460.52 | 1.0000 | 2.4e-256 | Bacteria/Proteobacteria |
| YOL095C | 75.58 | 1.0000 | 1.5e-33 | Bacteria/Firmicutes |
| YMR090W | 89.27 | 1.0000 | 1.7e-39 | Bacteria/Firmicutes |
| YIL164C | 160.65 | 1.0000 | 1.7e-70 | Bacteria/Proteobacteria |
| YIL165C | 54.67 | 1.0000 | 1.8e-24 | Bacteria/Proteobacteria |
| YNR057C | 73.72 | 1.0000 | 9.6e-33 | Bacteria/Proteobacteria |

**Supplementary table 2**. Gene candidates for horizontal transfer from inside of fungi in *Saccharomyces cerevisiae* via high-throughput identification.

| **Gene** | **Bitscore** | **Out_pct** | **HGT index** | **Donor taxonomy** |
| --- | --- | --- | --- | --- |
| YIL166C | 563.9 | 0.9361 | 0.5253 | Fungi/Pezizomycotina |
| YJL030W | 209.1 | 0.8083 | 0.5296 | Fungi/Pezizomycotina |
| YFL056C | 293.1 | 0.8854 | 0.6831 | Fungi/Pezizomycotina |
| YKR094C | 253.8 | 0.8794 | 0.9937 | Fungi/Pezizomycotina |
| YJR153W | 377.9 | 0.9218 | 0.5385 | Fungi/Pezizomycotina |
| YKL190W | 243.8 | 0.8217 | 0.7135 | Fungi/Pezizomycotina |
| YCR063W | 194.5 | 0.8244 | 0.5941 | Fungi/Taphrinomycotina |
| YNL331C | 589.7 | 0.8712 | 0.7744 | Fungi/Pezizomycotina |
| YDL243C | 522.3 | 0.8689 | 0.7847 | Fungi/Pezizomycotina |
| YCR107W | 498.0 | 0.8779 | 0.6855 | Fungi/Pezizomycotina |
| YJR155W | 457.2 | 0.8678 | 0.7830 | Fungi/Pezizomycotina |
| YNR073C | 547.4 | 0.9474 | 0.5305 | Fungi/Pezizomycotina |
| YOL165C | 190.3 | 0.8519 | 0.6748 | Fungi/Pezizomycotina |
| YOR339C | 216.9 | 0.8519 | 0.6625 | Fungi/Taphrinomycotina |
| YHR129C | 423.7 | 0.8275 | 0.5421 | Fungi/Pezizomycotina |
| YOR378W | 671.0 | 0.8867 | 0.6539 | Fungi/Pezizomycotina |
| YLR162W-A | 105.9 | 0.9161 | 0.7474 | Fungi/Ustilaginomycotina |
| YGL255W | 407.9 | 0.8404 | 0.5532 | Fungi/Pezizomycotina |
| YLR154W-A | 135.6 | 0.9149 | 1.0000 | Fungi/Agaricomycotina |
| YDR538W | 275.4 | 0.8920 | 0.5739 | Fungi/Pezizomycotina |
| YIL148W | 253.8 | 0.8794 | 0.9937 | Fungi/Pezizomycotina |
| YJL213W | 369.8 | 0.8631 | 0.5511 | Fungi/Pezizomycotina |
| YPR094W | 156.8 | 0.8189 | 0.6471 | Fungi/Pezizomycotina |
| YOR094W | 252.7 | 0.8442 | 0.6863 | Fungi/Pezizomycotina |
| YLL063C | 588.6 | 0.9655 | 0.6184 | Fungi/Pezizomycotina |
| YDR539W | 559.3 | 0.8852 | 0.5402 | Fungi/Agaricomycotina |
| YEL070W | 547.4 | 0.9474 | 0.5305 | Fungi/Pezizomycotina |
